# Nationwide Cross-Sectional Study of the Association between Knee Pain and Weight Change: Analysis of the Korea National Health and Nutrition Examination Survey (KNHANES 2013–2015)

**DOI:** 10.3390/ijerph18105185

**Published:** 2021-05-13

**Authors:** Sungwoo Choi, Sangun Nah, Hae-Dong Jang, Seung-Hee Cheon, Ji-Eun Moon, Sangsoo Han

**Affiliations:** 1 Department of Emergency Medicine, Soonchunhyang University Bucheon Hospital, Bucheon 14584, Korea; csw3613@naver.com (S.C.); potter325@naver.com (S.N.); 2 Department of Orthopedic Surgery, Soonchunhyang University Bucheon Hospital, Bucheon 14584, Korea; khaki00@schmc.ac.kr; 3 Department of Orthopedic Surgery, VHS Medical Center, Seoul 05368, Korea; csh717@nate.com; 4 Department of Biostatistics, Clinical Trial Center, Soonchunhyang University Bucheon Hospital, Bucheon 14584, Korea; moon6188@schmc.ac.kr

**Keywords:** knee joint, weight gain, obesity

## Abstract

The knee is a hinge joint that provides stability and control, which are essential in daily life. Obesity is a major cause of knee pain and its incidence continues to increase worldwide. In this study, we analyzed Korea National Health and Nutrition Examination Survey data on the general population, and showed an association between weight change and knee pain. A total of 22,948 participants were enrolled; those under the age of 50 and those who did not answer the questions about knee pain or weight change were excluded. In all, 8480 patients were analyzed, 7001 (82.56%) of whom indicated that they did not have knee pain, versus 1479 (17.44%) who did experience knee pain. Multivariate regression analysis was performed to analyze the association between knee pain and weight change. With full adjustment for covariates, weight gain per se (OR 1.37; *p* = 0.002), and gains of 3–6 kg (OR 1.28; *p* = 0.029) and ≥6 kg (OR 1.62; *p* = 0.012), showed significant associations with knee pain. This cross-sectional study confirmed a significant association between knee pain and weight change. Therefore, when evaluating patients with knee pain, it is necessary to evaluate weight gain.

## 1. Introduction

The knee is a hinge joint that has to provide stability and control during weight bearing and the performance of flexion and rotation [1]. These functions are essential for daily life. Knee pain has various causes, affects many aspects of daily life, reduces quality of life, and causes functional impairment and loss of independence [2,3]. The American Rheumatology Society criteria for a clinical diagnosis of knee osteoarthritis (OA) include an age ≥50 years. Approximately 25% of the general population aged ≥50 years experiences pain due to knee OA [4,5].

Risk factors for knee pain include female sex, low education, depression, obesity, old age, previous knee injury, and performance of work that places a strain on the knees [6,7]. The incidence of obesity continues to increase worldwide, and a third of the world’s population is now classified as overweight or obese [8]. Obesity imposes a considerable burden on the musculoskeletal system, particularly on the weight-bearing lower limbs [9]. In many previous studies, there was a significant association between obesity and knee pain, or diseases such as knee OA, for which obesity is an established risk factor [2,10,11]. Both simple obesity and weight gain are associated with knee pain [2]. However, no study has reported the association of knee pain with weight gain in the general population.

The purpose of this study is to investigate the association between weight change and knee pain in the Korean general population aged ≥50 years by using the Korea National Health and Nutrition Examination Survey (KNHANES) data.

## 2. Materials and Methods

### 2.1. Data Collection

Data obtained from the KNHANES of 2013 (VI-1), 2014 (VI-2), and 2015 (VI-3) were analyzed. The KNHANES is an annual survey conducted by the Korea Centers for Disease Control and Prevention (KCDC) to investigate the health and nutritional status of the Korean population. It uses a clustered, multistage, stratified, and random sampling method, where 8000–10,000 individuals are selected annually from about 4000 households based on gender, region of residence, and age. Each year, different participants are selected, i.e., the same participants are not monitored over time. The survey is conducted by medical staff and specialists, and is divided into three parts: health surveys, physical examinations, and dietary surveys [12]. We analyzed the data of the participants enrolled in VI-1–3; those aged ≥50 years who answered the questions about knee pain were included (the KNHANES VI-1–3 did not assess knee pain in individuals aged <50 years), while participants who did not respond to those questions, or to questions on weight change, were excluded.

### 2.2. Definitions of Knee Pain and Weight Change

The question on knee pain was as follows: “Did you have knee joint pain for more than 30 days in the last 3 months?” Individuals who answered “Yes” were included in our study.

The question on weight change was as follows: “Have there been any changes in your weight during the last year? If so, how much weight did you lose or gain?” Answers were categorized as “No change”, weight loss of 3–6 or ≥6 kg, or weight gain of 3–6 or ≥6 kg.

### 2.3. Demographic, Social, and Health-Related Variables

Through questionnaires and interviews, data on age, sex, height, weight, body mass index (BMI), duration of sleep, smoking status, alcohol consumption, education level, occupation, household income, degree of stress, physical activity, and medical comorbidities were obtained.

BMI was calculated by dividing weight (kg) by the height squared (m^2^). According to the Asian-Pacific region criteria, <18.5 was classified as underweight, 18.5–24.9 as normal weight, and ≥25 as obese [13]. The duration of sleep was evaluated by the following question: “How many hours do you usually sleep a day?” Smoking status was classified as non-smoker/ex-smoker or current smoker. Alcohol consumption was classified as ≤1 drink/month, 2–3 drinks/week, or ≥4 drinks/week. Education level was classified as elementary school (≤6 years), middle school (7–9 years), high school (10–12 years), or university (≥13 years). Employment was classified as unemployed (student, housewife, etc.), office work; sales and services; agriculture, forestry, and fishery; or manual labor [14]. Household income was divided into quartiles. Degree of subjective stress was classified as none, mild, moderate, or severe. Physical activity was defined as at least 2.5 h per week of moderate-intensity aerobic exercise or at least 1.25 h per week of high-intensity aerobic exercise [15]. As comorbidities, data were obtained regarding hypertension, diabetes mellitus, dyslipidemia, stroke, myocardial infarction, angina, arthritis, asthma, and malignancy.

### 2.4. Statistical Analyses

The participants were divided into two groups according to the presence or absence of knee pain. Student’s *t*-test was used to compare continuous variables, and chi-squared test to compare categorical variables. The association between knee pain and weight change was analyzed by multiple logistic regression according to weight change. Odds ratios (ORs) were calculated along with 95% confidence intervals (CI). We constructed three models to explore mediators and confounders potentially associated with knee pain. Model 1 was unadjusted as a crude model. Model 2 was adjusted for age and sex which were known as risk factors for knee pain [6,7]. Model 3 was adjusted for age, sex and other environmental factors such as obesity, duration of sleep, smoking, alcohol consumption, educational level, occupation, household income, degree of stress, and physical activity. We chose to adjust the variables that were known to be associated with knee pain in previous studies [5,16], and/or were shown to be statistically different between two groups with or without knee pain in our study. Statistical analyses were performed using IBM SPSS Statistics software (ver. 26.0; IBM Corp., Armonk, NY, USA). *p*-values < 0.05 were considered statistically significant. Sampling weights were applied to prevent bias.

## 3. Results

**Figure 1:**
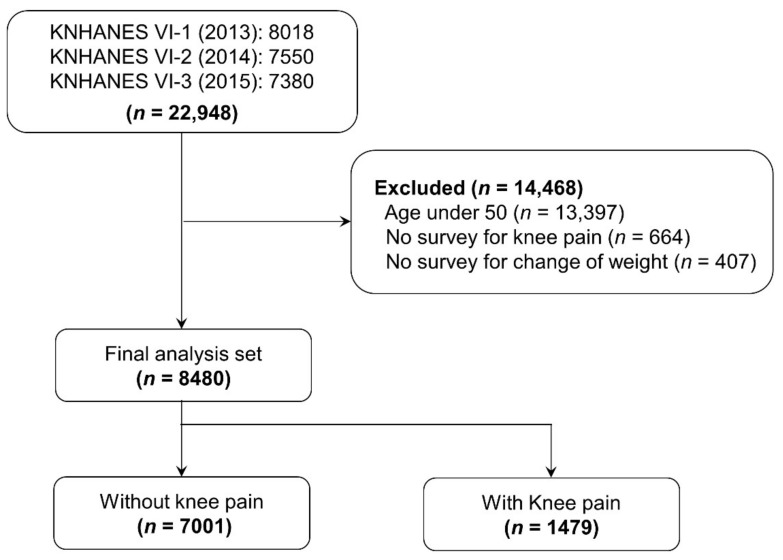
Flow chart of the study populations of the 2013–2015 Korea National Health and Nutrition Examination Surveys (KNHANES VI-1-3, respectively).

A total of 8018 people participated in KNHANES VI-1 (2013), compared to 7550 in VI-2 (2014) and 7380 in VI-3 (2015) (total of 22,948 people). Among them, 13,397 participants aged <50 years, 664 who did not answer questions on knee pain, and 407 participants who did not answer questions on weight change were excluded. A total of 8480 participants were analyzed, of whom 7001 (82.56%) indicated that they did not have knee pain, compared to 1479 (17.44%) who did experience knee pain (Figure 1).

### 3.1. General Characteristics According to Presence of Knee Pain

A total of 946 (63.96%) subjects with knee pain and 5053 (72.18%) without knee pain reported no weight change (*p* < 0.001). The numbers of subjects reporting weight loss and weight gain in the two groups were 255 (17.24%) and 1011 (14.44%), and 278 (18.8) and 937 (13.38), respectively (both *p* < 0.001). The numbers of subjects reporting weight loss of 3–6 and ≥6 kg in the two groups were 194 (16.18%) and 811 (13.38%), and 59 (4.92%) and 198 (3.27%), respectively (both *p* < 0.001). The numbers reporting weight gain of 3–6 and ≥6 kg were 216 (17.65%) and 781 (13.04%), and 62 (5.07%) and 156 (2.6%), respectively (both *p* < 0.001). There were significant differences between the two groups in age, sex, height, weight, BMI, duration of sleep, alcohol consumption, education level, occupation, household income, degree of stress, and physical activity (all *p* < 0.001) (Table 1).

### 3.2. Association between Knee Pain and Weight Change

**Figure 2:**
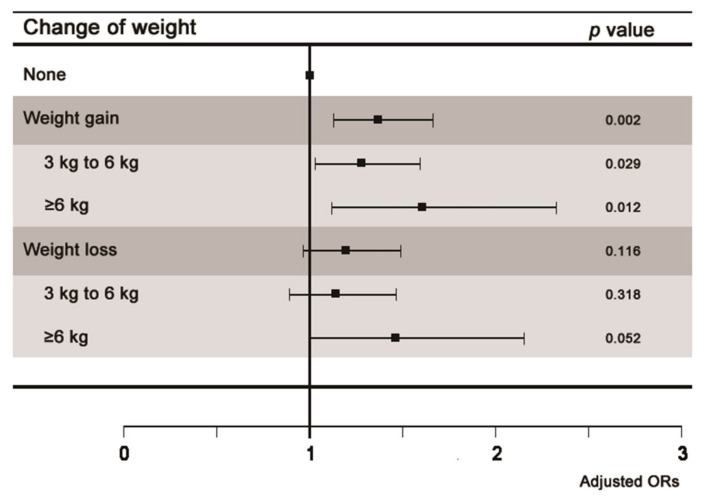
Odds ratios of weight change in participants with knee pain. Model 1, unadjusted; Model 2, adjusted for sex and age; Model 3, adjusted for age, sex, and other factors such as obesity, duration of sleep, smoking, alcohol consumption, educational level, occupation, household income, degree of stress, physical activity, and comorbidities. CI, confidence interval; OR, odds ratio.

Multivariate regression analysis was performed to analyze the association between knee pain and weight change. In the fully adjusted model (Model 3), weight gain (OR 1.37; 95% CI 1.12–1.67; *p* = 0.002) showed a significant association with knee pain, whereas weight loss (OR 1.2; 95% CI 0.96–1.49; *p* = 0.116) did not (Table 2). In the multivariate regression analysis on the amount of weight gain and loss, for the fully adjusted model, weight loss of 3–6 kg (OR 1.14; 95% CI 0.88–1.76; *p* = 0.318) and ≥6 kg (OR 1.47; 95% CI 0.99–2.17; *p* = 0.052) showed no significant association with knee pain, while weight gain of 3–6 kg (OR 1.28; 95% CI 1.03–1.59; *p* = 0.029) and ≥6 kg (OR 1.62; 95% CI 1.11–2.35; *p* = 0.012) showed a significant association with knee pain (Table 3 and Table 4). The adjusted ORs and 95% CIs of the three models for the association between knee pain and weight change are shown in Figure 2.

## 4. Discussion

By analyzing KNHANES data on the general population of Korea, we confirmed an association between knee pain and weight change. We analyzed a total of 22,948 people, excluding those aged <50 years and those who did not answer questions on knee pain or weight change. Finally, 8480 participants were analyzed. After adjusting for all factors that may affect knee pain, we confirmed a significant elevation in the risk of knee pain with weight gain. Increases in body weight of 3–6 and ≥6 kg were associated with ≥1.2 and ≥1.6-fold increased risks of knee pain.

In previous studies, being overweight, weight gain, and obesity were significantly associated with knee pain [2,10,11,17], and were also risk factors for knee OA [18]. In this study, greater weight gain was more significantly associated with knee pain. Most previous studies were about obesity or BMI [10,11,17]. Although there are studies showing an association between weight change and knee pain [2,18], to the best of our knowledge no studies on the association between weight change and knee pain have been conducted in the general population.

**Figure 3:**
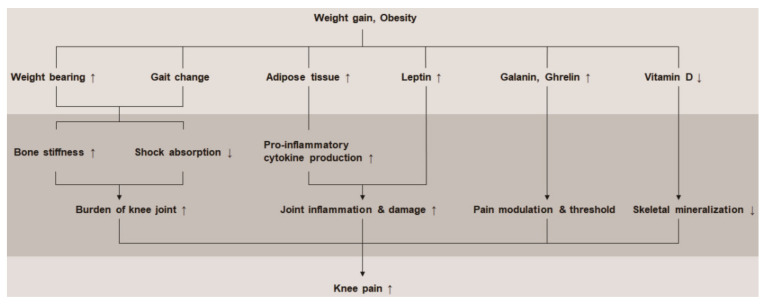
Theory regarding the pathophysiological effects of weight gain and obesity on knee pain.

An increase in weight increases the burden on the knee joint, which is weight-bearing [9]. It also causes a change in the biomechanical component of the gait, which affects the knee joint [19]. Repeated loading induces bone stiffness and decreases the duration of eccentric quadriceps tension associated with heel strike during gait, thereby reducing shock absorption and adversely affecting the knee joint [19,20]. In addition, weight gain causes changes in body composition, thus increasing fat mass. These adipose tissues cause dysregulation of cytokine production, overproduction of pro-inflammatory cytokines [21,22], and an increase in leptin, which promotes joint inflammation and damage [23,24]. Obesity also affects the levels of hormones and peptides, such as ghrelin and galanin, and is thought to be involved in pain modulation [25,26]. A lack of vitamin D is more common in obese people, which leads to poor skeletal mineralization and joint pain [27,28] (Figure 3). These biomechanical and neuroendocrinological factors are consistent with the results of this study. The OR for knee pain significantly increased with the body weight increase in our multiple regression analyses, even after adjusting for all covariates.

Previous studies have shown that weight loss improves symptoms and prognosis in knee pain and OA patients [29,30,31]. However, since this study did not measure pain over time, it was not possible to determine whether weight loss leads to pain relief. However, weight loss is not implicated in knee pain. In model 3, fully adjusted for all other environmental factors, weight loss was not related to knee pain, unlike in model 1 and 2. Among the environmental factors, arthritis (OR 5.57; 95% CI 4.60–6.74; *p* < 0.001) was found to be the key factor that caused knee pain and weight loss together.

When treating patients with knee pain, physicians need to understand the anatomical or organic basis of the problem, and must carefully check for weight gain. Using a simple questionnaire that can be easily used in the clinic, this study confirmed a significant association between weight gain and knee pain.

There were several limitations to this study. First, the KNHANES data were not collected for this study. The KNHANES data were originally collected to examine the health and nutritional status of the Korean population. Thus, a large number of participants (14,468) were excluded from this study. However, as 92.6% (13,397) of these people were excluded for being under 50 years of age, our results could be considered to be representative of the Korean general population aged ≥50 years. Second, because it used a cross-sectional design, causality could not be inferred in the relationship between weight change and knee pain. However, because the general population was analyzed via a clustered, multistage, random sampling method, sampling and selection biases were minimized. Third, as the survey was relatively basic, a detailed evaluation of the association between knee pain and weight change was not possible. An objective measure of knee pain, such as the Western Ontario and McMaster Universities Osteoarthritis Index, could be used to analyze pain severity and disability [32]. In addition, since weight changes were not actually measured, instead being obtained through the questionnaire, there was a risk of bias. Fourth, the KNHANES only assesses knee pain in participants aged ≥50 years. Fifth, we did not consider the effect of sarcopenia on knee pain, although sarcopenia is known to be one of the risk factors [33]. Sixth, an influence of ethnicity on the findings cannot be excluded, so studies in other countries are needed.

## 5. Conclusions

This cross-sectional study analyzed nationwide health survey data, and confirmed an association between knee pain and weight change; the greater the weight gain, the more significant the association with knee pain. Thus, when evaluating patients with knee pain, it is necessary to determine the degree of weight gain.

## Figures and Tables

**Table 1:** General participant characteristics according to knee pain.

	Without Knee Pain (*n* = 7001)	With Knee Pain (*n* = 1479)	*p*-Value
Age, year	63.37 ± 8.89	67.04 ± 8.94	<0.001
Sex, *n* (%)			<0.001
Male	3257 (46.52)	352 (23.8)	
Female	3744 (53.48)	1127 (76.2)	
Height (cm)	160.09 ± 8.76	155.48 ± 8.36	<0.001
Weight (kg)	61.68 ± 10.39	60.09 ± 9.87	<0.001
Obesity (BMI), *n* (%)			<0.001
Underweight (<18.5)	188 (2.69)	25 (1.69)	
Normal (18.5–24.9)	4379 (62.61)	810 (54.84)	
Obese (≥25)	2427 (34.7)	642 (43.47)	
Duration of sleep, h	6.62 ± 1.47	6.4 ± 1.72	<0.001
Smoking status, *n* (%)			<0.001
Non-/Ex-smoker	5912 (84.45)	1331 (89.99)	
Current smoker	1089 (15.55)	148 (10.01)	
Alcohol consumption, *n* (%)			<0.001
None	2655 (37.92)	742 (50.17)	
≤1 drink/month	1762 (25.17)	369 (24.95)	
2 drinks/month to 3 drinks/week	1990 (28.42)	279 (18.86)	
≥4 drinks/week	594 (8.48)	89 (6.02)	
Education level, *n* (%)			<0.001
≤6 year	2505 (37.85)	966 (65.71)	
7–9 year	1166 (17.62)	236 (16.05)	
10–12 year	1837 (27.76)	211 (14.35)	
≥13 year	1110 (16.77)	57 (3.88)	
Occupation, *n* (%)			<0.001
Unemployed (student, housewife, etc.)	3161 (47.76)	908 (61.73)	
Office work	708 (10.7)	52 (3.54)	
Sales and services	743 (11.23)	108 (7.34)	
Agriculture, forestry, and fishery	1200 (18.13)	219 (14.89)	
Simple labor	806 (12.18)	184 (12.51)	
Household income, *n* (%)			<0.001
Low	1852 (26.61)	668 (45.38)	
Low-moderate	1867 (26.82)	369 (25.07)	
Moderate-high	1584 (22.76)	238 (16.17)	
High	1658 (23.82)	197 (13.38)	
Degree of stress, *n* (%)			<0.001
None	1803 (25.82)	243 (16.56)	
Mild	3952 (56.59)	760 (51.81)	
Moderate	986 (14.12)	353 (24.06)	
Severe	243 (3.48)	111 (7.57)	
Change in weight, *n* (%)			<0.001
No change	5053 (72.18)	946 (63.96)	
Weight loss	1011 (14.44)	255 (17.24)	
Weight gain	937 (13.38)	278 (18.8)	
Amount of weight loss, *n* (%)			<0.001
None	5053 (83.36)	946 (78.9)	
3–6 kg	811 (13.38)	194 (16.18)	
≥6 kg	198 (3.27)	59 (4.92)	
Amount of weight gain, *n* (%)			<0.001
None	5053 (84.36)	946 (77.29)	
3–6 kg	781 (13.04)	216 (17.65)	
≥6 kg	156 (2.6)	62 (5.07)	
Physical activity, *n* (%)	1999 (30.16)	317 (21.54)	<0.001
Comorbidities, *n* (%)			
Hypertension	2517 (35.95)	712 (48.14)	<0.001
Diabetes mellitus	961 (13.73)	286 (19.34)	<0.001
Dyslipidemia	1497 (21.38)	454 (30.7)	<0.001
Stroke	282 (4.03)	92 (6.22)	<0.001
Myocardial infarction	104 (1.49)	34 (2.3)	0.0329
Angina	210 (3)	82 (5.54)	<0.001
Arthritis	1056 (15.88)	889 (60.11)	<0.001
Asthma	205 (2.93)	103 (6.96)	<0.001
Malignancy	200 (2.86)	49 (3.31)	0.3899

Values are expressed as the mean ± SD or number (proportion). BMI, body mass index. *p* < 0.05 was taken to indicate statistical significance.

**Table 2:** Association between weight change and knee pain: results of multiple logistic regression.

Wt. Change	Model 1			Model 2			Model 3		
	OR	95% CI	*p*-Value	OR	95% CI	*p*-Value	OR	95% CI	*p*-Value
No change	1			1			1		
Wt. loss	1.48	1.23–1.78	<0.001	1.49	1.23–1.81	<0.001	1.20	0.96–1.49	0.116
Wt. gain	1.54	1.30–1.82	<0.001	1.57	1.32–1.87	<0.001	1.37	1.12–1.67	0.002

Wt.; weight, OR; odds ratio, CI; confidence interval. Model 1 was unadjusted odds ratio. Model 2 was adjusted by age, and sex. Model 3 was fully adjusted by age, sex, and other environmental factors such as obesity, duration of sleep, smoking, alcohol consumption, educational level, occupation, household income, degree of stress, physical activity, and comorbidities.

**Table 3:** Association between amount of weight loss and knee pain using multiple logistic regression.

Amount of Wt. Loss	Model 1			Model 2			Model 3		
	OR	95% CI	*p*-Value	OR	95% CI	*p*-Value	OR	95% CI	*p*-Value
No Wt. loss	1			1			1		
3 kg to 6 kg	1.38	1.11–1.70	0.003	1.41	1.13–1.75	0.002	1.14	0.88–1.76	0.318
≥6 kg	1.95	1.39–2.75	<0.001	1.85	1.28–2.67	0.001	1.47	0.99–2.17	0.052

Wt.; weight, OR; odds ratio, CI; confidence interval. Model 1 was the unadjusted odds ratio. Model 2 was adjusted by age and sex. Model 3 was fully adjusted by age, sex, and other environmental factors such as obesity, duration of sleep, smoking, alcohol consumption, educational level, occupation, household income, degree of stress, physical activity, and comorbidities.

**Table 4:** Association between amount of weight gain and knee pain using multiple logistic regression.

Amount of Wt. Gain	Model 1			Model 2			Model 3		
	OR	95% CI	*p*-Value	OR	95% CI	*p*-Value	OR	95% CI	*p*-Value
No Wt. gain	1			1			1		
3 kg to 6 kg	1.39	1.16–1.68	<0.001	1.42	1.14–1.73	<0.001	1.28	1.03–1.59	0.029
≥6 kg	2.33	1.67–3.24	<0.001	2.34	1.66–3.28	<0.001	1.62	1.11–2.35	0.012

Wt.; weight, OR; odds ratio, CI; confidence interval. Model 1 was the unadjusted odds ratio. Model 2 was adjusted by age and sex. Model 3 was fully adjusted by age, sex, and other environmental factors such as obesity, duration of sleep, smoking, alcohol consumption, educational level, occupation, household income, degree of stress, physical activity, and comorbidities.

## Data Availability

The data are available from the KCDC and Prevention database on the following webpage: https://knhanes.kdca.go.kr/knhanes/sub03/sub03_02_05.do (accessed on 5 April 2021). The data are available via this web page to anyone who meets the appropriate qualifications.
